# UPR Induction Prevents Iron Accumulation and Oligodendrocyte Loss in *ex vivo* Cultured Hippocampal Slices

**DOI:** 10.3389/fnins.2018.00969

**Published:** 2018-12-18

**Authors:** Sinead Healy, Jill McMahon, Una FitzGerald

**Affiliations:** Galway Neuroscience Centre, School of Natural Sciences, National University of Ireland Galway, Galway, Ireland

**Keywords:** iron, unfolded protein response, UPR, tunicamycin, ferrocene

## Abstract

The accumulation of iron within the brain occurs in many chronic disorders including Alzheimer’s and Parkinson’s disease and multiple sclerosis. Outside the CNS, a link between levels of iron and the unfolded protein response has already been established. To determine if such a relationship operates in within the brain, we used our *ex vivo* hippocampal slice-based model of iron accumulation. Ferrocene addition caused accumulation of iron within slices and loss of oligodendrocytes, an effect that was partially inhibited when ferrocene and ER stressor tunicamycin (Tm) were added together. An upward trend (not found to be statistically significant) in the expression of UPR transcripts in response to ferrocene was demonstrated using real-time PCR, while a significant upregulation of mRNA for B cell immunoglobulin-binding protein (BiP) and C/EBP homologous binding protein (CHOP) occurred following exposure to Tm. *In silico* analysis revealed consensus DNA-binding sequences for UPR-associated transcription factors within the promoter regions of eight iron-regulatory genes. In addition, dual-staining for CHOP and oligodendrocyte transcription factor 2 (OLIG2) or Ionized calcium binding adaptor molecule 1 (Iba1) showed nuclear expression of CHOP in some oligodendrocyte-lineage cells in response to Tm or Tm+ferrocene, but CHOP was rarely found in microglia. Co-expression of UPR-associated activated transcription factor 6 (ATF6) was detected in the nuclei of some oligodendrocyte-lineage cells exposed to Tm alone, or to Tm and ferrocene, but rarely in microglia. These data highlight the therapeutic potential of targeting UPR-associated proteins when developing novel treatments for chronic brain disorders that are affected by dysregulated iron.

## Introduction

The unfolded protein response (UPR) is a ‘check and balance’ program activated upon disturbed homeostasis in the endoplasmic reticulum. Occasional but infrequent hints have documented an interplay between the UPR and iron metabolism (Ye and Connor, 2000; You et al., 2003; Oliveira et al., 2009; Vecchi et al., 2009). However, reports are few on the possible links between the UPR and iron within the brain (Liu and Connor, 2012; Rahman et al., 2017, 2018; Lumsden et al., 2018). A better understanding of this cross-talk might provide further clues regarding disease pathogenesis, given that aberrant iron metabolism and increased expression of markers of the UPR have been independently reported in several neurodegenerative diseases (Hetz and Mollereau, 2014), including multiple sclerosis (Stephenson et al., 2014), Alzheimer’s disease and Parkinson’s disease (Mercado et al., 2013; Ward et al., 2014). Researchers have exploited glial and neuronal mono- and dual-cultures *in vitro*, as well as *ex vivo* systems to investigate the regulation of iron within the brain [see (Healy et al., 2017) for comprehensive review of this topic]. As a foundation for future studies, the overall goal of this work was therefore to confirm and characterize the reciprocal relationship between UPR and iron homeostasis within our hippocampal slice-based model (Healy et al., 2016) of the accumulation of iron within the CNS.

## Methods

### Ethics Statement

The research described was approved by the Animal Care Research Ethics Committee Filing ID 12/FEB/04) and was carried out in accordance with European directive 2010/63/EU.

### Hippocampal Slice Culture and Reagent Exposure

Neonatal hippocampal slices were maintained as previously described (Healy et al., 2016).

Five μg/ml tunicamycin (Tm, T7765, Sigma-Aldrich, Dublin, Ireland) exposure was used to activate a UPR response, while iron loading was induced by a 12-h treatment with 1 μM ferrocene, a membrane-permeable compound that enables uptake of iron by cells (F408, Sigma-Aldrich, Dublin, Ireland). Slices were harvested immediately after iron loading.

### Analysis of RNA Transcripts

RNA was isolated from control and treated slice cultures using TRIreagent (T9424, Sigma-Aldrich, Dublin, Ireland) as per the manufacturer’s instructions. Following DNAse I treatment (18068-015, Thermofisher-Scientific, Dublin, Ireland) and cDNA synthesis using manufacturer protocol (1804014, Superscript II Reverse Transcriptase, Invitrogen, Dublin, Ireland), real-time PCR was accomplished using Applied Biosystems 4346906 MicroAmp^®^Fast Optical 96-Well Reaction Plate and Fast SYPR green master mix (applied Biosystems, 4385612) and the Step One real-time PCR System (Thermo-Fisher).

Unless otherwise indicated, oligonucleotide primer sequences (Sigma-Aldrich, Dublin, Ireland) used were designed using Primer3. Primer sequence details are summarized in the Supplementary Table 1. Results were quantified using the ΔΔCT method following standardization relative to β-actin as an internal control.

### Tissue Viability

Tissue viability was measured as described in Healy et al. (2016), using a lactate dehydrogenase (LDH) and MTT [3-(4,5-Dimethylthiazol-2-yl)-2,5-Diphenyltetrazolium Bromide] assay.

### Immunohistochemistry and Imaging

Tissue was fixed and stained as previously described (Healy et al., 2016). Briefly, slices were fixed in 4% paraformaldehyde (PFA) for 30 min followed by three washes in PBS and then blocked and permeabilised for 1 h with 10% normal goat serum and 0.4% Triton X-100 in PBS. Subsequently, the slices were incubated with primary antibody (for single and dual labeling) in 2.5% NGS (or 2.5% horse serum) and 0.1% Triton X-100 in PBS for 48 h at 4°C and then washed three times for 15 min before incubation with the appropriate secondary antibody at 4°C overnight. Source information and dilutions used for all antibodies is available in the Supplementary Materials. Slice cultures were mounted in Vectashield containing diamino-2-phenylindole (DAPI) (H-1200, Vector Laboratories, Peterborough, United Kingdom) to allow visualization of nuclei. Negative “no primary antibody” controls were included. Slides were stored in the dark at 4°C until imaged. All samples were imaged on a laser scanning confocal microscope (Olympus Fluoview 1000) using a 40 × oil-immersion lens (numerical aperture of 1.3).

### *In silico* Analysis of UPR Transcription Factor Binding Sites

The ALGGEN PROMO algorithm^1^ was used to determine if any UPR transcription factor consensus DNA binding sequences were present within the promoter regions of genes that encode iron homeostasis regulators. Only hits that had a dissimilarity index <15% and a random expectation <0.5 were included in this analysis.

### Statistical Analysis

Data was analyzed using Prism 6 (GraphPad Software). All measurements were expressed as mean ± standard error of the mean (SEM) unless otherwise indicated. Normality was assessed using the Shapiro-Wilk test. Statistical analyses were carried out using Student’s *t*-test, Mann-Whitney test or an ANOVA with Dunnet post-test, as appropriate, and as indicated in figure legends. Differences were considered statistically significant if *P*<0.05.

## Results

### UPR Transcript Expression in Cultured Slices Exposed to Ferrocene Remains Unchanged

**FIGURE 1:**
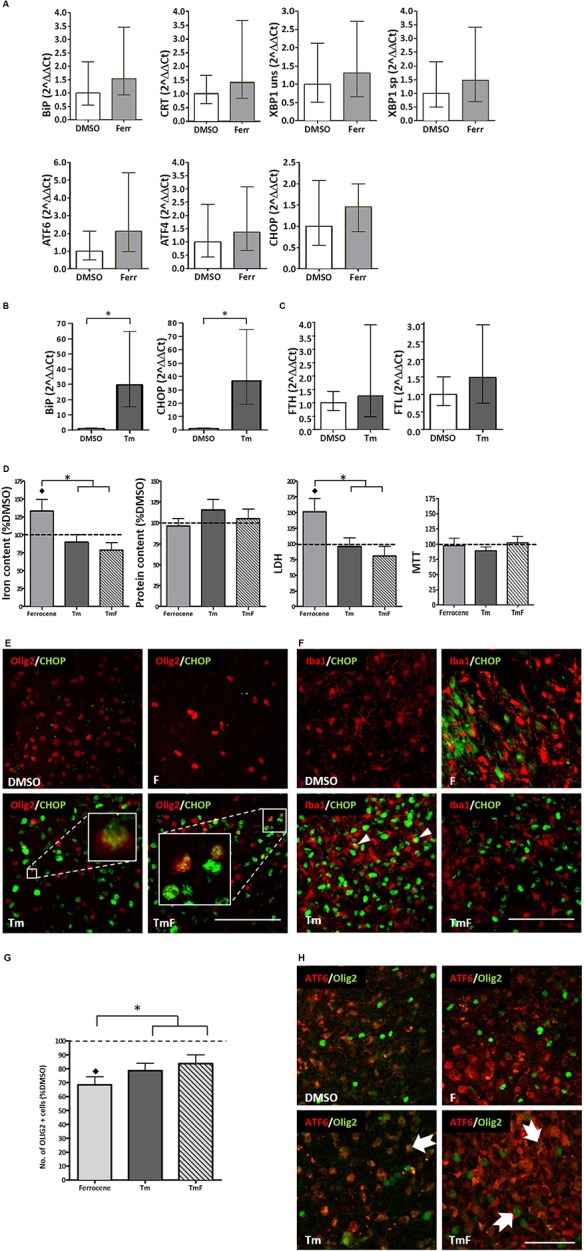
Cross-talk between regulators of the UPR and iron homeostasis. **(A)** Real-time PCR analysis of RNA samples derived from cultured slices treated for 12 h with 1 μM ferrocene. An upward trend in the expression of BiP, CRT, XBP1, ATF6, ATF4, and CHOP was detected (*n* = 3 experimental replicates). **(B)** Increased BiP and CHOP transcripts detected following 12 h treatment with 5 μg/ml Tm (*n* = 3). Data is expressed as mean ± 95% confidence interval following completion of three experimental repeats. ^∗^denotes *p*<0.05. **(C)** Upward trend in the expression of FTL and FTH transcripts detected following 12 h Tm treatment (5 μg/ml, *n* = 2 experimental repeats). **(D)** Effect of Tm exposure (5 μg/ml, 12 h) on iron and protein content and on tissue viability (*n* = 3 experimental replicates). Cultures were loaded with 1 μM ferrocene **(F)**, 5 μg/ml Tm or both treatments concurrently (TmF). Data is expressed as percentage of DMSO control, which is indicated by the dashed line. Values are mean + SEM from three experiments. ♢ denotes a significant difference (*p*<0.05) when compared with DMSO and ^∗^denotes significant difference between F-treated slices and those treated with Tm or TMF (*p*<0.05). **(E)** CHOP expression (green) was detected within the nuclei of olig2-positive cells (red) in hippocampal slices treated with Tm or TmF (lower panels), but was not seen within the Olig2-positive population of control or F-treated slices (upper panels). Scale bar = 100 μm **(F)** When cultured slices were dual-labeled using antibodies to Iba1 (red) and CHOP (green, RH panels), CHOP was not found in DMSO-exposed slices but was seen in non-Iba1-positive slices treated with F or TmF and occasionally in Iba1-positive nuclei in slices treated with Tm alone (white arrowheads). Scale bar = 100 μm**. (G)** The significant loss of oligodendrocyte-lineage cells caused by exposure to F is partially reversed when slices are co-treated with Tm or TmF (*n* = 2 experimental replicates). ♢denotes a significant difference (*p*<0.05) when compared with DMSO; ^∗^denotes a significant difference between F-treated slices and those treated with Tm or TMF (*p*<0.05). **(H)** Cytoplasmic expression of ATF6 (red) detected in the non-olig2-expressing cells of DMSO- and F-treated hippocampal slices (top panels). Treatment with Tm or TmF led to nuclear expression of ATF6 within olig2-positive cells (green, arrows). Scale bar = 50 μm.

Real-time PCR analysis of changes in the transcripts for B-cell immunoglobulin binding protein (BiP), calreticulin (CRT), X-box binding protein 1 (XBP1), activated transcription factor 6 (ATF6), activated transcription factor and C/EBP homologous binding protein (CHOP) revealed an approximate 1.4–2.1-fold increase, which was not found to be statistically significant following three replicate experiments (Figure 1A).

### UPR Induction Does Not Increase Expression of Iron Storage Protein Transcripts

The ability of cultured hippocampal slices to mount a response to ER stress was confirmed when a significant increase in BiP and CHOP transcription (approximately 30-fold for both), was detected following a 12-h treatment with 5 μg/ml tunicamycin (Tm, Figure 1B). Tm inhibits N-glycosylation of proteins trafficked through the ER (Foufelle and Fromenty, 2016) hence causing a build-up of mis-folded proteins and ER stress. However, Tm was not found, after two experimental repeats, to cause a significant increase in the expression of ferritin light chain (FTL) or ferritin heavy chain (FHL) transcripts (Figure 1C).

### UPR Activation Prevents Ferrocene-Induced Iron Accumulation and Glial Perturbations

In line with our previous findings (Healy et al., 2016), 1 μM ferrocene produced a 1.3-fold increase in the levels of iron in slice cultures when compared to vehicle (Figure 1D). Similarly, we confirmed that ferrocene-induced a significant 1.5-fold increase in LDH release, and, once again, did not report any iron-induced loss in metabolic activity as assessed by MTT assay (Figure 1D). Interestingly, concurrent exposure to 5 μg/ml Tm (TmF) resulted in amelioration in iron accumulation and toxicity (Figure 1D). TmF treatment led to a significant reduction in iron content and a significant decrease in LDH release, when compared with ferrocene alone, but not when compared with the DMSO control. In keeping with our data relating to FTL and FTH transcriptional expression, Tm exposure alone did not lead to a significant difference in iron content. Tm treatment alone also had no effect on tissue viability when compared to DMSO control conditions. There were no significant differences in metabolic activity as measured using an MTT assay, or in protein content between groups (Figure 1D).

### UPR Transcription Factor Expression in Glia Following Tm and Iron Exposure

Classically, CHOP is upregulated in response to phosphorylation of PERK and is frequently exploited as a marker of activation of the UPR. Immunofluorescent staining demonstrated an absence of CHOP expression in control and ferrocene-treated slice cultures, but robust upregulation of CHOP in Tm-treated tissue. Dual immunofluorescent staining confirmed CHOP expression in Olig2-positive cells within Tm-treated slices, which appeared to increase when slices were co-exposed to Tm and Ferrocene (Figure 1E). Less frequently, CHOP was found to be present in microglia within cultured slices exposed to Ferrocene, Tm or both (Figure 1F). Similar to our prior finding of significantly reduced numbers of microglial endpoints, following slice treatment with F, we also found, following two experimental repeats, fewer end-points after F treatment, which was rescued when tissue was treated with TmF (data not shown). ATF6 is the second major regulator of the UPR whose protein expression we analyzed by immunofluorescence within cultured control and treated slices. While non-nuclear ATF6 was detected in control DMSO-treated cultures, an increase in nuclear ATF6 staining was observed following treatment with Tm, F, or a combination of both reagents (not shown).

In our previous study, we reported a significant loss of oligodendrocyte-lineage cells following iron loading (Healy et al., 2016). This result was confirmed here (Figure 1G). Intriguingly, slice exposure to Tm or TmF reversed this loss, to the extent that Olig2-lineage cells responded by increasing their number significantly (Figure 1G). Dual immunofluorescence was then used to determine if the localization of ATF6 expression within Olig2-positive cells was affected by the same conditions. Our images showed that while nuclear (active) ATF6 was not detectable within DMSO-treated Olig2-lineage cells, it was present in Tm-, F- or TmF-treated cultures (Figure 1H).

### *In silico* Analysis of Putative Transcription Factor Binding Sites Within Promoter Regions of Iron-Regulatory Genes

Using the ALGGEN PROMO algorithm to predict consensus transcription factor (TF) DNA binding sequences, we identified a number of putative binding sites for UPR-associated transcription within the promoter regions of iron homeostasis genes (Table 1). None of the TFs were found to be commonly expressed on all the promoter regions examined. Activated transcription factor 3 (ATF3) and CCAAT-enhancer-binding protein alpha (C/EBP-α) consensus-binding sequences were found on six of the eight genes (Table 1). Consensus sequences for XBP1, ATF and ATF2 were found within two promoter regions; the CREB consensus sequence was found on three genes and C/EBP-β was not found on any. Therefore, there was not a consistent pattern of transcription factor hits across the different genes (Table 1).

**Table 1:** Putative binding sites of UPR transcription factors in the promoters of genes encoding iron homeostasis proteins.

Gene	Factor	Start	End	Dissimilarity	String	RE Equally	RE Query
**Hepc**	ATF3 [T01313]	23	60	6.74	tgacacaa	0.082	0.062
	ATF3 [T01313]	445	45	13.48	tgctgtca	0.164	0.132
	XBP-1 [T00902]	379	384	6.47	atgccc	0.292	0.318
	XBP-1 [T00902]	448	453	1.58	tgtcat	0.220	0.231
**FTH**	ATF3 [T01313]	497	504	10.12	tgacaaaa	0.222	0.225
	ATF3 [T01313]	521	528	10.12	tgacttca	0.222	0.225
	C/EBPα [T00105]	301	307	3.55	accaatc	0.137	0.225
	C/EBPα [T00105]	425	431	7.47	tgcaatg	0.273	0.290
	C/EBPα [T00105]	527	533	2.44	cattgag	0.273	0.278
	C/EBPα [T00105]	571	577	2.37	aattggg	0.273	0.279
	C/EBPα [T00105]	1063	1069	8	tccaatg	0.137	0.141
**FTL**	XBP-1 [T00902]	494	499	6.48	atgagg	0.401	0.434
	C/EBPα [T00105]	211	217	5.58	gtcaatt	0.401	0.283
	C/EBPα [T00105]	754	760	5.78	gccaata	0.401	0.283
**TfR1**	C/EBPα [T00105]	1609	1615	1.22	tattgag	0.345	0.420
	C/EBPα [T00105]	1822	1828	0	gattgag	0.345	0.334
	CREB [T00163]	2347	2355	3.08	tgacgtttg	0.086	0.091
**Tf**	C/EBPα [T00105]	1410	1416	4.56	gacaatc	0.280	0.270
	ATF3 [T01313]	1040	1047	8.31	gtatgtca	0.316	0.311
	ATF3 [T01313]	1397	1404	10.12	tgacctca	0.456	0.445
	ATF3 [T01313]	1887	1894	0	ttacgtca	0.035	0.033
	CREB [T00163]	1886	1894	2.66	attacgtca	0.070	0.069
	ATF-2 [T00167]	1886	1895	4.17	attacgtcaa	0.040	0.037
	ATF [T00051]	1887	1898	8.419	ttacgtcaacag	0.089	0.087
**FPN**	ATF3 [T01313]	2376	2383	8.31	atatgtca	0.455	0.429
	ATF3 [T01313]	2745	2752	8.31	atatgtca	0.455	0.429
	ATF3 [T01313]	3237	3244	3.37	tgacataa	0.151	0.179
	C/EBPα [T00105]	759	765	5.024	cacaatc	0.404	0.370
	C/EBPα [T00105]	777	783	3.55	gattgga	0.404	0.533
	C/EBPα [T00105]	1433	1439	4.56	gattggc	0.404	0.367
**Cp**	C/EBPα [T00105]	74	80	8	cattgga	0.405	0.505
	C/EBPα [T00105]	935	941	0.54	cacaatc	0.405	0.361
	C/EBPα [T00105]	1421	1427	1.22	tattgag	0.405	0.361
	C/EBPα [T00105]	1430	1436	0.54	gattggg	0.405	0.361
	ATF3 [T01313]	932	939	6.74	tgacacaa	0.456	0.519
**DMT1**	C/EBPα [T00105]	2	8	3.55	accaatc	0.463	0.566
	CREB [T00163]	3116	3124	2.664	tgacgtgat	0.116	0.119
	ATF3 [T01313]	2716	2723	3.372	tgacataa	0.173	0.192
	ATF-2 [T00167]	3115	3124	4.17	ttgacgtgat	0.065	0.075
	ATF [T00051]	3112	3123	5.46	gcattgacgtga	0.048	0.047


*Summary of the putative binding site of the UPR-associated transcription factors in the gene sequence of an iron-homeostasis molecule with a dissimilarity index <15% and random expectation <0.5. Transcription factors tested: C/EBP-beta, C/EBP-α, CREB, XBP1, ATF, ATF3, ATF-2, and ATF1.*

## Discussion

In an attempt to elucidate whether or not cross-talk between iron-regulatory proteins and ER stress, we exploited our rat hippocampal slice-based model of iron accumulation. Following slice exposure to Tm, F or both, real-time PCR and fluorescent immunohistochemistry yielded the following primary findings: (1) Tm exposure does not affect the level of iron in hippocampal slices, but it triggers a UPR that appears to prevent iron overload and possibly ameliorates toxic effects of iron on oligodendrocytes and microglia; (2) iron overload causes an upward trend in the levels of transcripts encoding the major markers of the UPR, although this was not statistically significant; (3) the observation that Tm treatment led to no significant change in the expression of FTL or FTH transcripts was surprising, since the promoter regions of eight genes that encode iron-regulatory proteins, including FTL and FTH, contain putative binding sites for ER stress-associated transcription factors; (4) transcription factor ATF6 is activated following exposure of cultured hippocampal slices to Tm, F and TmF; (5) Activated ATF6 is detectable within the Tm- and TmF-treated oligodendrocycte population but rarely in similarly treated microglia.

While intriguing, it remains unclear how concurrent iron accumulation and UPR activation, demonstrated here, leads to the ameliorated accumulation of iron. Outside the brain, the main description of the intersection between the UPR and iron metabolism is the action of CHOP on hepcidin (via modulation of C/EBPα binding and/or through CREBH) in the liver and spleen (Oliveira et al., 2009; Vecchi et al., 2009) although ER stress associated chaperones calreticulin and BiP have also been implicated in iron UPR cross-talk [reviewed in (Oliveira et al., 2011)]. More recently, an increase in hepcidin expression leading to accumulated iron within the brain of rats subjected to sub-arachnoid hemorrhage, was found to be regulated by CHOP (Zhao et al., 2018).

Our observation that CHOP immunofluorescence appears to be increased in Olig-2 positive cells in slices that were co-exposed to Tm and Ferrocene compared with only Tm is consistent with these studies and it hints at the potential therapeutic benefit of UPR proteins induced by Tm to protect against iron-induced toxicity within oligodendrocyte lineage cells. However, further work is required to elucidate or reasonably speculate on such a mechanism.

## Author Contributions

SH carried out the PCR. SH and JM completed the tissue staining. JM and UF conceived and oversaw research described. SH, JM, and UF wrote the manuscript.

## Conflict of Interest Statement

The authors declare that the research was conducted in the absence of any commercial or financial relationships that could be construed as a potential conflict of interest.

## Footnotes

1 http://alggen.lsi.upc.es/
