# Supplementary material for: UPR Induction Prevents Iron Accumulation and Oligodendrocyte Loss in ex vivo Cultured Hippocampal Slices
Source: Front Neurosci. 2018 Dec 18;12:969. doi: 10.3389/fnins.2018.00969 (PMC6305600; doi:10.3389/fnins.2018.00969)
Supplement: Supplementary file 1 [file Table_1.docx]

Supplementary Material

UPR Induction Prevents Iron Accumulation and Oligodendrocyte Loss in ex vivo Cultured Hippocampal Slices

Sinead Healy^1^, Jill McMahon^1^, Una FitzGerald^1*^

*** Correspondence:**

Corresponding Author una.fitzgerald@nuigalway.ie

Supplementary Table 1: PCR Primer Sequence Information. Unless otherwise noted, all sequences were designed using Primer3.

| Gene | Accession No. | |  | Forward (5’-3’) | Reverse (5’-3’) |
| --- | --- | --- | --- | --- | --- |
|  | | **Iron-associated molecules** | | | |
| FTL | nm_022500.4 | |  | ctcctcaagttgcagaacgaac | gttttaccccactcatcttg |
| FTH | nm_012848.2 | |  | atccccacttatgtgacttc | cttgtcaaagagatattctgcc |
|  | | **UPR-associated molecules** | | | |
| ATF4 | nm_024403.2 | |  | tcagacaccggcaaggag | gtggccaaaagctcatctg |
| ATF6 | nm_001107196.1 | |  | ggaccaggtggtgtcagag | gacagctctgcgctttgg |
| BiP | nm_013083.2 | |  | cctattcctgcgtcggtgtatt | ggttggacgtgagttggttc |
| CHOP | nm_024134.2 | |  | gaaatcgagcgcctgaccag | ggaggtgatgccaacagttca |
| CRT | nm_022399.2 | |  | agcagttcttggacggagatg | tgtttggattcgacccagc |
| XBP1s | nm_001004210 | |  | gagtccgcagcaggtgc | ggtccaacttgtccagaatgc |
| XBP1uns | nm_001004210 | |  | cagactacgtgcgcctctg | cttctgggtagacctctggg |
|  | | **Housekeeping genes** | | | |
| ßactin ^‡^ | nm_031144.3 | |  | cacactgtgcccatctatga | ccatctcttgctcgaagtct |

‡ The primer sequences were taken from the following publication: (Kraskiewicz and FitzGerald, 2011);

All primers were optimised to ensure specificity and optimal efficiency.

Reference for supplementary material

Kraskiewicz, H., and FitzGerald, U. (2011). Partial XBP1 knockdown does not affect viability of oligodendrocyte precursor cells exposed to new models of hypoxia and ischemia in vitro. *J Neurosci Res* 89(5)**,** 661-673. doi: 10.1002/jnr.22583.
